# Community-based outreach associated with increased health utilization among Navajo individuals living with diabetes: a matched cohort study

**DOI:** 10.1186/s12913-020-05231-4

**Published:** 2020-05-25

**Authors:** Calvin Franz, Sidney Atwood, E. John Orav, Cameron Curley, Christian Brown, Letizia Trevisi, Adrianne Katrina Nelson, Mae-Gilene Begay, Sonya Shin

**Affiliations:** 1 Eastern Research Group, Inc., Lexington, MA USA; 2 Department of Global Health and Social Medicine, Harvard Medical School, Boston, MA USA; 3 Department of Biostatistics, Harvard T.H. Chan School of Public Health, Boston, MA USA; 4 Division of Global Health Equity, Brigham and Women’s Hospital, Boston, MA USA; 5 Department of Global Community Health and Behavioral Sciences, Tulane School of Public Health and Tropical Medicine, 1440 Canal Street, New Orleans, LA USA; 6 Navajo Nation Community Health Representative Outreach Program, Navajo Nation Department of Health, Window Rock, AZ USA; 7 Harvard Medical School, Boston, MA USA

**Keywords:** Health utilization, Health disparities, Diabetes mellitus, Community health workers, Community health representatives, Clinic-community linkages, American Indian, Navajo

## Abstract

**Background:**

Navajo community members face high rates of diabetes mellitus and other chronic diseases. The Navajo Community Health Representative Outreach Program collaborated with healthcare providers and academic partners to implement structured and coordinated outreach to patients living with diabetes. The intervention, called Community Outreach and Patient Empowerment or COPE, provides home-based health coaching and community-clinic linkages to promote self-management and engagement in healthcare services among patients living with diabetes. The purpose of this study was to evaluate how outreach by Navajo Community Health Representatives (“COPE Program”) affected utilization of health care services among patients living with diabetes.

**Methods:**

De-identified data from 2010 to 2014 were abstracted from electronic health records at participating health facilities. In this observational cohort study, 173 cases were matched to 2880 controls. Healthcare utilization was measured as the number of times per quarter services were accessed by the patient. Changes in utilization over 4 years were modeled using a difference-in-differences approach, comparing the trajectory of COPE patients’ utilization before versus after enrollment with that of the control group. The model was estimated using generalized linear mixed models for count outcomes, controlling for clustering at the patient level and the service unit level.

**Results:**

COPE enrollees showed a 2.5% per patient per quarter (pppq) greater increase in total utilization (*p* = 0.001) of healthcare services than non-COPE enrollees; a 3.2% greater increase in primary care visits (*p* = 0.024); a 6.3% greater increase in utilization of counseling and behavioral health services (*p* = 0.013); and a 9.0% greater increase in pharmacy visits (*p* <  0.001). We found no statistically significant differences in utilization trends of inpatient, emergency room, specialty outpatient, dental, laboratory, radiology, or community encounter services among COPE participants versus control.

**Conclusions:**

A structured intervention consisting of Community Health Representative outreach and coordination with clinic-based providers was associated with a modest increase in health care utilization, including primary care and counseling services, among Navajo patients living with diabetes. Community health workers may provide an important linkage to enable patients to access and engage in clinic-based health care.

**Trial registration:**

NCT03326206, registered 10/31/2017, retrospectively registered.

## Background

In recent decades, American Indian/Alaska Native (AI/AN) communities have experienced rising rates of cardiovascular disease [1], with a prevalence of cardiovascular disease among AI/AN nearly two-fold higher than the overall US population [2]. Rates of diabetes mellitus (DM) have also dramatically increased over the past few decades in many AI/AN communities. Mortality due to diabetes is 2.5 to 3.5 times higher among AI/AN populations than the United States mortality rate across all races [3]. The cost of diabetes care is also considerable; O’Connell, et al., estimated that diabetes care accounted for 37% of all adult treatment expenses in one Indian Health Service (IHS) facility [4].

The Navajo Nation experiences numerous challenges to improving cardiovascular disease care. Many patients living on the Navajo reservation struggle with chronic disease management due to access barriers to regular healthcare services. Primary care services are challenged by chronic understaffing of healthcare professionals and limited resources [5]. Extreme geographic distances and lack of transportation make appointment attendance and medication refills difficult. Furthermore, patients face barriers to cross-cultural communication with their IHS providers [5]. Health education is often suboptimal due to a lack of patient educational materials appropriate to AI/AN populations [6].

There is growing advocacy for integrating community health workers (CHWs) within the U.S. healthcare delivery system [7]. The Patient Protection and Affordable Care Act identifies community health workers as an important part of the care team for delivery system reform and authorizes funding for CHWs in medically underserved communities [8]. The Institute of Medicine has recommended that CHWs should be integrated into multidisciplinary teams as an approach to eliminate health disparities [7, 9], and the Centers for Disease Control and Prevention has recommended integral inclusion of CHWs for diabetes management [10]. Similarly, the Agency for Healthcare Research and Quality conducted a systematic review of CHW interventions and found that community health workers can improve health outcomes among underserved populations; however, they noted that methodological limitations of many of the studies limited the ability to draw strong conclusions across a variety of clinical conditions [11]. Overall, there is growing momentum to incorporate CHWs as integral members of the care team particularly when caring for underserved populations, which requires community outreach and cultural competency [12]. To better address workforce shortages and create patient-centered medical homes [8], there is an urgent need for data regarding the impact of robust, real-world models of CHWs when they are integrated into the clinic team and function at the top of their skill set.

The Community Outreach and Patient Empowerment (COPE) intervention seeks to address health disparities in AI/AN communities by strengthening the role of CHWs and building stronger linkages between community and clinic-based providers. COPE activities focus on building synergy between IHS quality improvement activities and the Navajo Nation Community Health Representative (CHR) Program within a socio-ecological framework [13, 14]. CHRs are tribal CHWs and have a long history in tribal healthcare systems. For the purposes of this manuscript, the term CHW refers to the broader healthcare profession which includes CHRs. By strengthening coordination and collaboration among inter-professional teams involving IHS providers and CHRs, COPE reaches out to high-risk patients with uncontrolled cardiovascular disease risk factors to promote healthy lifestyles, facilitate access to the healthcare delivery system, and enhance self-efficacy of patients, family members and CHRs themselves. The intervention expands the role of CHRs by providing skills, knowledge, and networks to deliver effective support to high-risk individuals.

We have studied the effectiveness of the COPE intervention and found it improves glycosylated hemoglobin (HbA1c) control for diabetic patients [15]. In this paper, we examine the impact of the COPE program on healthcare utilization by individuals living with diabetes on Navajo Nation. We hypothesized that the intervention would result a greater increase in healthcare utilization compared with control patients, without an increase in hospitalization or emergency department services.

## Methods

### Study setting and population

This observational cohort study took place in Navajo Nation from 2010 through 2014. Navajo Nation has 332,129 members and covers 27,000 mile^2^ in parts of Arizona, Utah, and New Mexico [16]. The Navajo Area Indian Health Service (NAIHS) is one of 12 regional administrative units of the national IHS system. The NAIHS is divided into eight sub-regional “Service Units”, comprised of a total of 6 hospitals, 7 health centers, and 15 health stations. Separate from Indian Health Services, the tribe oversees its own health programs under the auspices of the Navajo Nation Department of Health, including the Navajo CHR Program. Formally established in 1968, the Navajo CHR Program includes nearly 100 CHRs who offer crucial services for patients and families including health education and in-home health assessments. Each Service Unit has between six and 18 CHRs, each of whom is assigned to specific communities, called Chapters.

### Program intervention

COPE was started in 2010 as collaboration between the Navajo CHR Program, Brigham and Women’s Hospital, and the NAIHS. This program has been implemented in all eight Service Units of the Navajo Area, and more than 650 patients have been enrolled to date. The “COPE intervention” is comprised of three inter-related strategies designed to strengthen existing community outreach and linkage to clinic-based care. The intervention focuses on three activities: 1) training CHRs to ensure proficiency in health topics and build skills in behavior change techniques such as motivational interviewing and goal-setting; 2) developing patient coaching materials that CHRs can then use in the home; and 3) facilitating greater connection and communication between the patient and clinic-based team. Centers for Disease Control and Prevention recommendations for strengthening the role of CHWs include 1) clear definitions regarding the scope of activities, 2) rigorous training standards, 3) increased clinician awareness about the role of CHWs, and 4) enhanced professional support networks for CHWs [17]. COPE addresses all of these recommendations through standardized CHR training, close supervision, quality control of CHR activities, and integration of CHR activities into the primary care teams in IHS facilities.

### Participant selection

Although individuals living with any chronic condition may enroll in COPE, the vast majority have Type 2 diabetes mellitus, and, as described above, diabetes is a serious chronic health condition prevalent in Navajo Nation. Therefore, our prospective cohort study of COPE enrollees included COPE enrollees who: (1) have been diagnosed with diabetes, (2) received care in one of the participating Service Units, and (3) had at least one baseline measure of HbA1c prior to enrolling in COPE. The study encompassed six of the eight Service Units in Navajo Nation that had implemented COPE; we excluded two Service Units because of low COPE enrollment at one site, and use of a different electronic health record system at the other. We abstracted data for adults with an ICD-10 diagnosis of diabetes mellitus from the IHS resource and patient management system (RPMS). RPMS is an electronic health record that is used by the majority of IHS facilities for routine clinical care; each facility maintains an individual RPMS database with administrative and clinical data [18]. In addition to clinical diagnoses, we abstracted laboratory tests, vital signs, medications, and healthcare utilization data from RPMS.

A matching algorithm was generated to identify patients seen at multiple sites and to identify COPE patients within the database. Once patients were identified across sites and COPE cases were captured, the dataset was de-identified. Within this dataset, we matched COPE participants to non-COPE patients based on age (+/− 5 years), gender, primary health facility, hemoglobin HbA1c (+/− 1 point) and systolic blood pressure (+/− 10 mmHg) at baseline (i.e. 3 months prior to the date of COPE enrollment). The matching process is presented in detail in Trevisi et al. [17]

### Study outcomes and confounding factors

Our primary study outcome was the frequency with which patients are hospitalized, visit outpatient clinics, or otherwise use healthcare resources. IHS service unit utilization is primarily organized around clinics, and each healthcare encounter in RPMS lists the clinic visited by the patient. We used the RPMS clinic variable as the basis for the frequency of utilization analysis. With approximately 120 types of “encounters” (because “clinic” may also include miscellaneous types of utilization that are not, strictly speaking, a clinic visit, such as telephone call, chart review, and outpatient use of an inpatient treatment room, for example, we chose to use the broader term “type of encounter”), we grouped utilization into seven broad categories: community encounters, counseling/behavioral, dental, emergency, inpatient, primary care, and specialty care. Of note, community encounters included other community-based services – such as public health nurse visits and school visits – but did not include CHR visits.

Each type of encounter reported in RPMS was counted as one utilization incident for the purposes of this analysis. For inpatient services, the primary data point was the presence of a DRG code indicating the patient was hospitalized. However, we also included encounter listings for labor and delivery and for observation as inpatient utilization. Table 1 presents the encounters listed and their grouping for the purposes of the utilization analysis. We excluded from the analysis utilization such as telephone calls and telemedicine because the frequency with which these were reported suggested they were inconsistently recorded. Chart review was also excluded because it did not represent a separate, distinct encounter for the patient.

**Table 1:** Types of Encounters as Reported in RPMS and Categorization for Utilization Analysis

Category for Analysis	Category for Analysis
Reported Encounter Type [a]	Reported Encounter Type
**Community encounters**	**Specialty outpatient (cont.)**
Home Care	Chronic Disease
School	Colposcopy
**Counseling/behavior**	Complementary Medicine
Alcohol and Substance	Day Surgery
Behavioral Health	Dermatology
Day Treatment	Diabetic (including Footcare and Retinopathy)
Diabetes Education	Dialysis
Education Classes	Dietary
General Preventive	Endocrinology
Group Services	ENT
Medical Social Services	Family Planning
Mental Health	High Risk
Telebehavioral Health	Medication Therapy Management
Tobacco Cessation Clinic	Nephrology
Wellness	Neurology
** Dental**	Obesity
**Emergency**	Ob/Gyn
Emergency Medicine	Ophthalmology
Triage	Optometry
Urgent Care	Orthopedic
**Inpatient**	Pain Management
Labor and Delivery	Physical Therapy/Occupational Therapy
Observation	Plastic Surgery
Hospitalization (if DRG listed) [b]	Podiatry
**Primary Care Outpatient**	Postpartum
Elder Care	Pulmonology
Family Practice	Radiation Exposure Screening
General	Rehabilitation
Immunization	Respiratory Care
Internal Medicine	Rheumatology
Pediatric	Speech Pathology/Speech Therapy
Well Child	Sports Medicine
**Specialty Care Outpatient**	STD
Anesthesiology	Surgical
Anticoagulation Therapy	Teen Clinic
Audiology	Traditional Medicine
Cancer Screening	Urology
Cardiology	Women’s Health Screening
Chest and TB	Wound Care

[a] For the purposes of this table, some clinics reported as separate in RPMS have been combined for brevity (e.g. Physical Therapy/Occupational Therapy)

[b] Visits are also categorized as inpatient if no clinic is reported but a DRG is reported

A second outcome was the utilization of lab and radiology services by counting the individual number of lab or radiology tests ordered, identified using Current Procedural Terminology (CPT) codes. The use of pharmacy services was measured using the number of medications prescribed.

Our outcomes of interest were the total number healthcare utilizations by type of encounter and intervention group in each quarter. We also aggregated over encounter types to measure the frequency of total healthcare clinic utilization for each patient, but excluding lab, radiology, and pharmacy utilization.

### Community participation and ethical considerations

Our study protocols were developed and interpreted collaboratively with two stakeholder groups. The COPE Advisory Group established in 2012 is comprised of local physicians, nurses, program leaders, information technology specialists, Navajo Nation Department of Health program directors, and CHR supervisors and the Community Health Advisory Panel (CHAP), established in 2013, which includes COPE participants, their relatives, and CHRs. The COPE Advisory Group and Community Health Advisory Panel meet quarterly and provided suggestions on how to group health services, ensuring that study findings could be disseminated in a comprehensible manner to patients and families, and providing interpretive feedback on results.

For this study, Community Health Advisory Panel participants requested additional data on utilization of traditional medicine services. We shared that visits to Traditional Medicine services were included as Specialty Outpatient care and accounted for 476 of 82,803 (0.57%) of Specialty Outpatient visits. COPE Advisory Group members suggested a sensitivity analysis excluding community encounters (e.g. joint home visits between public health nurses and CHRs), in case CHRs visits may have been included in this group.

This study was approved by Partners Healthcare Institutional Review Board (2012P001069) and the Navajo Nation Human Research Review Board (NNR-11.150 T). In order to abstract data, study staff obtained necessary certification and training required by the Indian Health Services Rules of Behavior under the auspices of Collaborative Agreements with participating sites (NV-CA-10-0011, NA-CA-13-0013, NA-CA-11-0062).

### Statistical approach

We used a generalized linear mixed regression model for count outcomes, assuming a Poisson distribution for the outcome variable using a log-link to assess the differences in the frequency of healthcare utilization between COPE and non-COPE patients. The analysis was implemented with the SAS PROC GLIMMIX, and performed using SAS 9.3 (SAS Institute, Cary, North Carolina).

We used random effects to account for patient correlation over time, and within-site correlation at the service unit. We also adjusted for covariates with potential influences on utilization that exist at the time of the intervention: age, gender, language, primary care physician, and the following diagnoses: essential hypertension, major depression disorder, alcohol abuse, major cardiovascular disease (defined as at least one of the following diagnoses: acute myocardial infarction, coronary artery bypass surgery, coronary angioplasty, peripheral arterial disease, abdominal aortic aneurysm, carotid artery disease, cerebrovascular disease), and dyslipidemia.

In the model, we measured the outcome of interest as the number of encounters by encounter type that each participant had in each period, as well as the covariates listed above to adjust for differences between patients. The regression model measured the change (or trend) in utilization over time distinguishing the pre-intervention trend from the post-intervention trend, and utilization by COPE patients versus non-COPE patients. The model was parameterized so that the regression coefficients for utilization directly measure the difference in COPE patient utilization relative to non-COPE patients both before and after enrollment in COPE. Thus, we were most concerned with the value and statistical significance of the regression coefficient that measured the difference in utilization trend between the control group and the COPE group following the intervention. First, if it did not differ significantly from the post-intervention utilization trend of the control group, then statistically there was no difference between any post-intervention change that might have occurred to the control group and the COPE group. Even if a change occurred in the utilization trend, because the control group did not experience the COPE intervention, we could not attribute whatever change occurred in the COPE group to the COPE intervention. Second, if the COPE post-intervention coefficient was statistically significant and greater than zero, COPE patient utilization of health resources would have increased relative to control group patients; if it was statistically significant and less than zero, COPE patient utilization of health resources would have decreased relative to the control group. The model is presented in further detail in the technical appendix.

## Results

The study covered 17 quarters of healthcare utilization: 2 years (8 quarters) of utilization prior to the intervention, the quarter in which the patient enrolled in COPE, and 2 years following the intervention. Among the 28,813 adult patients with an ICD-10 diagnosis of DM in the database, a total of 173 COPE patients met study inclusion criteria, and were matched to 2880 control patients (10.1% of all non-COPE patients) comprising the cohort for evaluating healthcare utilization, the same cohort that was used to evaluate clinical endpoints [15].

Table 2 presents the baseline characteristics of the COPE and non-COPE cohorts. The majority of patients were between 56 and 85 years of age, and approximately two-thirds of both the COPE intervention and control groups were female. Almost 58% of COPE clients reported as primary language their Native language and not English, compared with 40.9% of the matched controls. The majority of individuals had both essential hypertension (65.3% among COPE clients, 68.3% among controls) and major cardiovascular disease (approximately 71% for both COPE clients and controls). In contrast, the reported prevalence of mental health disorders were low: depression was documented among 14.5 and 9.8% among COPE patients and controls, while alcohol use disorders were observed among 6.4% versus 2.7% of COPE versus control patients, respectively.

**Table 2:** Baseline Characteristics of Study Cohort [a]

	COPE *N* = 173	NON-COPE *N* = 2880
**Patient Characteristic**	**N (%)**	**N (%)**
**Age**		
25–40 y	7 (4.1)	46 (1.6)
41–55 y	33 (19.1)	610 (21.2)
56–70 y	74 (42.8)	1436 (49.9)
71–85 y	54 (31.2)	764 (26.5)
≥ 86 y	5 (2.9)	24 (0.8)
**Gender**		
Male	65 (37.6)	901 (31.3)
Female	108 (62.4)	1979 (68.7)
**Primary Language**		
Native American	100 (57.8)	1178 (40.9)
English	72 (41.6)	1700 (59.0)
Missing	1 (0.6)	2 (0.1)
**Primary Care Physician**		
Yes	146 (84.4)	2577 (89.5)
No	27 (15.6)	303 (10.5)
**Essential Hypertension**		
Yes	113 (65.3)	1966 (68.3)
No	60 (34.7)	909 (31.6)
Missing		5 (0.2)
**Major Depression Disorder**		
Yes	25 (14.5)	282 (9.8)
No	148 (85.6)	2593 (90.0)
Missing		5 (0.2)
**Alcohol abuse**		
Yes	11 (6.4)	77 (2.7)
No	162 (93.6)	2798 (97.2)
Missing		5 (0.2)
**Major Cardiovascular Disease**		
Yes	123 (71.1)	2050 (71.2)
No	50 (28.9)	825 (28.7)
Missing		5 (0.2)
**Dyslipidemia**		
Yes	80 (46.2)	1688 (58.6)
No	93 (53.8)	1187 (41.2)
Missing		5 (0.2)

[a] Characteristics evaluated at the closest available HbA1c measure before the enrollment date

Table 3 presents clinic utilization per patient per quarter (pppq) by type of encounter type and patient type. This demonstrates that, with the exception of dental care, the frequency of clinic utilization is consistently higher for COPE patients than for control patients both pre- and post-intervention.

**Table 3:** Healthcare Utilization by Type of Clinic Visited, Visits per Patient per Quarter, Pre- and Post-Intervention

		Intervention Patients				Control Patients			
Clinic		N^1^	Mean	Standard Deviation	95% CI	N^1^	Mean	Standard Deviation	95% CI
Community Encounters	Pre-	107	0.2466	0.3572	(0.1789, 0.3142)	497	0.122	0.3035	(0.0953, 0.1486)
	Post	86	0.7148	0.921	(0.5202, 0.9095)	413	0.3286	0.69	(0.2621, 0.3952)
Counseling/ Behavior	Pre-	133	0.2094	0.3824	(0.1445, 0.2744)	1548	0.1066	0.2535	(0.094, 0.1192)
	Post	129	0.5126	0.7001	(0.3918, 0.6335)	1595	0.3494	0.5511	(0.3223, 0.3764)
Dental	Pre-	69	0.1006	0.1555	(0.0639, 0.1373)	1394	0.1388	0.2204	(0.1272, 0.1503)
	Post	78	0.3286	0.4353	(0.232, 0.4252)	1417	0.4558	0.5582	(0.4268, 0.4849)
Emergency	Pre-	140	0.2957	0.5083	(0.2115, 0.3799)	2160	0.2246	0.375	(0.2088, 0.2404)
	Post	128	0.5796	0.7362	(0.4521, 0.7072)	2135	0.5148	0.5741	(0.4905, 0.5392)
Inpatient	Pre-	98	0.3715	1.0585	(0.1619, 0.581)	1469	0.2569	0.4078	(0.2361, 0.2778)
	Post	86	0.8735	1.1195	(0.6369, 1.1101)	1445	0.8056	1.0444	(0.7518, 0.8595)
Primary Care Outpatient	Pre-	159	0.5555	0.6957	(0.4473, 0.6636)	2541	0.4129	0.4029	(0.3972, 0.4285)
	Post	159	1.3736	1.0466	(1.2109, 1.5362)	2590	1.2159	0.8605	(1.1827, 1.249)
Specialty Care Outpatient	Pre-	161	0.4733	0.6338	(0.3753, 0.5712)	2672	0.3883	0.5745	(0.3665, 0.4101)
	Post	161	1.2309	1.362	(1.0205, 1.4413)	2635	0.9988	1.1018	(0.9567, 1.0409)
Total	Pre-	172	1.4983	1.2403	(1.313, 1.6837)	2851	1.092	0.8239	(1.0618, 1.1223)
	Post	173	4.3982	3.2826	(3.9091, 4.8874)	2870	3.3985	2.3366	(3.313, 3.484)

^1^ Number of patients with at least one visit in the 24 months prior to enrollment and at least one visit in the 24 months following enrollment

Over the 2-year (8 quarter) period following enrollment, COPE enrollees showed a modest statistically significant additional 2.5% quarterly increase compared to control patients, in total utilization of healthcare services (*p* = 0.0008; see Table 4). This increased utilization is primarily attributable to an additional: 3.2% increase in primary care outpatient visits (*p* = 0.024) and 6.3% increase in use of counseling/behavioral services (*p* = 0.0126). We also found a 9.0% increase in pharmacy utilization (*p* <  0.0001) relative to control patients. We found no statistically significant change in utilization of inpatient, emergency room, specialty outpatient, dental, laboratory, radiology, or community encounter services among COPE participants versus control.

**Table 4:** Percent Change in Trend of Average Healthcare Visits per Quarter by Type of Clinic Visited; 8 Quarters Pre- & Post-Intervention

Variable	Estimate [1]	95% CI	*p*-value
**Community Encounters**			
Non-COPE patient utilization trend, pre-intervention	0.9577	(0.9442, 0.9715)	< 0.0001
Difference, COPE versus non-COPE patient utilization trend, pre-intervention	0.9975	(0.9711, 1.0246)	0.8546
Difference, pre−/post-intervention utilization trend, non-COPE patients	1.0302	(1.0029, 1.0583)	0.0301
Difference, pre−/post-intervention utilization trend, COPE versus non-COPE patients	0.9960	(0.9462, 1.0484)	0.8791
**Counseling/Behavior**			
Non-COPE patient utilization trend, pre-intervention	1.0512	(1.0417, 1.0609)	< 0.0001
Difference, COPE versus non-COPE patient utilization trend, pre-intervention	0.9455	(0.921, 0.9707)	< 0.0001
Difference, pre−/post-intervention utilization trend, non-COPE patients	0.9171	(0.9027, 0.9316)	< 0.0001
Difference, pre−/post-intervention utilization trend, COPE versus non-COPE patients	1.0630	(1.0132, 1.1153)	0.0126
**Dental**			
Non-COPE patient utilization trend, pre-intervention	1.0295	(1.0209, 1.0381)	< 0.0001
Difference, COPE versus non-COPE patient utilization trend, pre-intervention	0.9836	(0.9444, 1.0243)	0.4236
Difference, pre−/post-intervention utilization trend, non-COPE patients	0.9690	(0.9552, 0.9829)	< 0.0001
Difference, pre−/post-intervention utilization trend, COPE versus non-COPE patients	1.0410	(0.9701, 1.1172)	0.2645
**Emergency**			
Non-COPE patient utilization trend, pre-intervention	0.9834	(0.9777, 0.9892)	< 0.0001
Difference, COPE versus non-COPE patient utilization trend, pre-intervention	0.9832	(0.9623, 1.0045)	0.1211
Difference, pre−/post-intervention utilization trend, non-COPE patients	0.9802	(0.9698, 0.9907)	0.0003
Difference, pre−/post-intervention utilization trend, COPE versus non-COPE patients	1.0073	(0.9669, 1.0493)	0.7290
**Inpatient**			
Non-COPE patient utilization trend, pre-intervention	1.0185	(1.012, 1.0251)	< 0.0001
Difference, COPE versus non-COPE patient utilization trend, pre-intervention	0.9804	(0.9564, 1.0051)	0.1186
Difference, pre−/post-intervention utilization trend, non-COPE patients	0.9856	(0.9748, 0.9966)	0.0102
Difference, pre−/post-intervention utilization trend, COPE versus non-COPE patients	1.0249	(0.9808, 1.071)	0.2734
**Primary Care Outpatient**			
Non-COPE patient utilization trend, pre-intervention	1.0241	(1.0199, 1.0283)	< 0.0001
Difference, COPE versus non-COPE patient utilization trend, pre-intervention	0.9806	(0.9654, 0.996)	0.0137
Difference, pre−/post-intervention utilization trend, non-COPE patients	0.9756	(0.9687, 0.9825)	< 0.0001
Difference, pre−/post-intervention utilization trend, COPE versus non-COPE patients	1.0319	(1.0041, 1.0605)	0.0243
**Specialty Care Outpatient**			
Non-COPE patient utilization trend, pre-intervention	1.0179	(1.0136, 1.0222)	< 0.0001
Difference, COPE versus non-COPE patient utilization trend, pre-intervention	1.0028	(0.987, 1.0188)	0.7333
Difference, pre−/post-intervention utilization trend, non-COPE patients	0.9701	(0.9629, 0.9774)	< 0.0001
Difference, pre−/post-intervention utilization trend, COPE versus non-COPE patients	1.0040	(0.9763, 1.0325)	0.7807
**Laboratory**			
Non-COPE patient utilization trend, pre-intervention	1.2005	(1.1916, 1.2094)	< 0.0001
Difference, COPE versus non-COPE patient utilization trend, pre-intervention	0.9806	(0.9543, 1.0077)	0.1602
Difference, pre−/post-intervention utilization trend, non-COPE patients	0.8008	(0.792, 0.8098)	< 0.0001
Difference, pre−/post-intervention utilization trend, COPE versus non-COPE patients	1.0218	(0.9799, 1.0655)	0.3122
**Pharmacy**			
Non-COPE patient utilization trend, pre-intervention	1.1347	(1.1282, 1.1413)	< 0.0001
Difference, COPE versus non-COPE patient utilization trend, pre-intervention	0.9245	(0.9067, 0.9427)	< 0.0001
Difference, pre−/post-intervention utilization trend, non-COPE patients	0.8073	(0.7996, 0.8151)	< 0.0001
Difference, pre−/post-intervention utilization trend, COPE versus non-COPE patients	1.0901	(1.0533, 1.1282)	< 0.0001
**Radiology**			
Non-COPE patient utilization trend, pre-intervention	1.0209	(1.0009, 1.0412)	0.0409
Difference, COPE versus non-COPE patient utilization trend, pre-intervention	1.0086	(0.9341, 1.0889)	0.8272
Difference, pre−/post-intervention utilization trend, non-COPE patients	0.9872	(0.9548, 1.0207)	0.4500
Difference, pre−/post-intervention utilization trend, COPE versus non-COPE patients	1.0136	(0.8948, 1.148)	0.8321
**Total 1 (including laboratory, pharmacy & radiology)**			
Non-COPE patient utilization trend, pre-intervention	1.0104	(1.0081, 1.0127)	< 0.0001
Difference, COPE versus non-COPE patient utilization trend, pre-intervention	0.9778	(0.97, 0.9856)	< 0.0001
Difference, pre−/post-intervention utilization trend, non-COPE patients	0.9830	(0.9791, 0.9868)	< 0.0001
Difference, pre−/post-intervention utilization trend, COPE versus non-COPE patients	1.0249	(1.0103, 1.0398)	0.0008
**Total 2 (excluding laboratory, pharmacy & radiology)**			
Non-COPE patient utilization trend, pre-intervention	1.0120	(1.0097, 1.0144)	< 0.0001
Difference, COPE versus non-COPE patient utilization trend, pre-intervention	0.9834	(0.9751, 0.9917)	0.0001
Difference, pre−/post-intervention utilization trend, non-COPE patients	0.9812	(0.9773, 0.9851)	< 0.0001
Difference, pre−/post-intervention utilization trend, COPE versus non-COPE patients	1.0205	(1.0052, 1.036)	0.0086

[1] The regression was performed as generalized linear mixed regression model for count outcomes, assuming a Poisson distribution for the outcome variable using a log-link. The regression results presented here are the exponentiated values of the specified coefficients to be directly interpretable as the percent change in utilization relative to enrollment in COPE

## Discussion

This study describes the impact on healthcare utilization of a programmatic clinical intervention to improve chronic disease management among individuals working with a CHR on Navajo Nation. The intervention increased the frequency with which COPE patients use NAIHS healthcare services by a modest but statistically significant amount. The effects were found in primary outpatient care, counseling and behavioral services, and pharmacy visits. The intervention has also resulted in a significant improvement in glycemic and lipid control 2 years following patient enrollment in the intervention [15].

Previous studies evaluating the impact of CHW interventions on healthcare utilization among individuals living with diabetes have been equivocal [19–21], likely due to the heterogeneity of CHW interventions themselves. Thus, the question of **how** the COPE intervention resulted in greater healthcare utilization has relevance for generalizability to other CHW programs. We believe COPE’s effectiveness reflects the socio-ecological approach to program design. At the patient level, we have described how patient self-efficacy is strengthened through emotional support, health coaching, and navigation support from CHRs to access care such as appointments and medication refills [22]. At the provider level, we have described how the intervention enhances CHRs’ ability to deliver standardized, evidence-based, culturally-tailored outreach to their patients and communicate more effectively with clinic providers about patient issues [23]. At the system level, we have demonstrated that the intervention strengthens community-clinic linkages, validating CHRs as members of the care team and increasing care coordination among clinic providers and CHRs [24]. These facets of the COPE intervention are collectively designed to enhance the role of CHRs as liaisons between the patient and their providers and to empower patients to engage in healthcare services. Both systems-level coordination and patient coaching emphasize primary care and self-management strategies over acute care, such as emergency room visits and hospitalizations. We speculate that both improved clinical outcomes and increased utilization of healthcare services have resulted from increased coordination and communication between CHRs and providers, as well as greater patient self-efficacy and motivation to actively seek healthcare.

This study of utilization leaves an important question outstanding: exactly what types of healthcare did they utilize? We have previously reported that we did not observe a significant difference in “clinical monitoring,” defined as HbA1c, LDL, and SBP measurements at least once in the past 12 months [15]. Nonetheless, it would be informative to assess adherence to American Diabetes Association standards of care [25]. We will examine that in a second paper based on the use of CPT codes, which will allow us to capture more nuance about utilization patterns. Using both measures provides a better picture of utilization: the increased frequency of visits is robust, since it is subject to relatively little measurement error, but somewhat crude: two primary care clinic visits are “equal” regardless of procedures performed during that visit. Conversely, although code-based reimbursement potentially allows us to compare and aggregate different types of utilization, it may be subject to more measurement error.

Study results should take into consideration that we included only COPE participants who received services at one of the participating sites during study period, relying on availability of routine medical records. Thus, findings may not be representative of other COPE patients who were not identified in the dataset, such as patients who received services elsewhere or not at all. Also, misclassification in diagnoses for specific health conditions as depression and alcohol abuse might affect the main results.

There are several limitations in the design of prospective cohort studies with matched controls. Identifying the study cohort is usually based on diagnostic criteria; in our case, we relied on CHRs to identify which individuals were enrolled in COPE Program. The central problem inherent in such studies is the selection of a comparable control group. The ideal control group would be a random sample from the same general population as the study cohort. We achieved this goal because our non-COPE controls were selected from the same population as the COPE patients. Although we matched non-COPE controls to COPE patients using socio-demographic and clinical characteristics, it was impossible to match for all baseline characteristics due to missing data and characteristics that were not recorded in health records. However, we were able to match more multiple non-COPE controls to each COPE patient to improve the statistical power of our study.

This study did not measure the differences in utilization of CHR services, which could not be collected using RPMS data. While outside the scope of this study, an audit of CHR patient encounter forms demonstrated that home visits (including travel time) to COPE participants versus non-participants took 7 minutes longer on average (35 versus 28 min). Given CHRs also visited COPE participants at least monthly, we estimate CHRs dedicated an additional 3.9 h per year for each COPE participant compared to other CHR clients. CHRs have provided feedback that COPE has contributed to a culture shift toward value-based care, with greater emphasis on clinical monitoring and health education. It is also likely that other CHR clients have benefited from the intervention in a less intensive manner, given that CHRs used flipcharts and behavior change skills as needed with all of their clients. Thus, our study might underestimate the impact of COPE since it attributes no intervention effect to non-COPE patients. Furthermore, to the extent that control patients are affected by, and in the same way as, COPE patients (e.g., increased frequency of use), our study will be less likely to find a statistically significant difference in post-intervention utilization because the difference will be smaller than if there was no COPE effect on controls.

## Conclusion

Our findings suggest that CHR outreach to individuals living with diabetes may increase patient utilization of outpatient healthcare services, without an increase in hospitalization or emergency room visits. We conclude that community health workers – when integrated with the clinic-based team – may be effective in increasing patient engagement with primary care services.

## Supplementary information

**Additional file 1.** Technical Appendix. Technical Appendix Table 1. Percent Change in Trend of Average Healthcare Visit per Quarter by Type of Clinic Visited; 8 Quarters Pre- & Post-Intervention; Complete Model Results [1].

## Data Availability

The datasets generated and/or analyzed during the current study are not publicly available because all data belong to the Navajo Nation per Navajo Human Research Review Board stipulations. Requests for data may be directed to the Navajo Human Research Review Board.

## Abbreviations

AI/AN: American Indian / Alaska Native
CHR: Community Health Representative
CHW: Community Health Worker
COPE: Community Outreach and Patient Empowerment
DM: Diabetes mellitus
HbA1c: Glycosylated hemoglobin
ICD-10: International Statistical Classification of Diseases and Related Health Problems, 10th edition
IHS: Indian Health Services
mm Hg: millimeters of mercury
NAIHS: Navajo Area Indian Health Services
pppq: Per patient per quarter
RPMS: Resource and patient management system
