# Supplementary material for: Community-based outreach associated with increased health utilization among Navajo individuals living with diabetes: a matched cohort study
Source: BMC Health Serv Res. 2020 May 25;20:460. doi: 10.1186/s12913-020-05231-4 (PMC7247176; doi:10.1186/s12913-020-05231-4)
Supplement: Supplementary file 1 — Additional file 1. Technical Appendix. Technical Appendix Table 1. Percent Change in Trend of Average Healthcare Visit per Quarter by Type of Clinic Visited; 8 Quarters Pre- & Post-Intervention; Complete Model Results [1]. [file 12913_2020_5231_MOESM1_ESM.docx]

**Technical Appendix**

This technical appendix presents the specification of the difference in differences model in greater detail and the complete regression results.

Conceptually, we measure the outcome of interest, Y_ijk_, as the number of encounters of type j (i.e., clinic type) that each participant i had at time k, β_0_ is the overall intercept. We used random effects to account for patient correlation over time (I_i_), and within-site correlation at each healthcare “service unit” (s_u_). Random effects follow a normal distribution.

Y_ijk_ = β_0_ + β_1_C_i_ + β_2_T1_k_ + β_3_C_i_*T1_k_ + β_4_T2_k_ + β_5_C_i_*T2_k_ + β_m_X_mi_ + … + β_u_X_ui_ + I_i_s_u_ + ε_ijk_

For participant i with encounter type j in period k: C_i_ (binary) indicates whether the patient is a COPE or non-COPE patient, and the coefficient for that variable, β_1_, measures the difference between the intercept for COPE and non-COPE patients.

We specified two time variables:

- T1_k_ = time from enrollment (TFE) and ranges -8 ≤ TFE ≤ 8
  - If -8 ≤ TFE < 0, then the patient is in the pre-enrollment period;
  - TFE = 0 is the period the patient enrolled in COPE;
  - If 1 ≤ TFE < 8, the the patient is post-enrollment.:

The coefficient β_2_ measures pre-enrollment utilization by non-COPE patients over time. The term C_i_*T1_k_ represents COPE patient enrollment status, and β_3_ measures the difference in pre-intervention utilization between COPE and non-COPE patients.

- T2_k_ = time past enrollment (TPE)
  - TPE = 0 if -8 ≤ TFE ≤ 0
  - TPE = TFE if 1 ≤ TFE ≤ 8

The coefficient β_4_ estimates the change in non-COPE utilization following enrollment. The effect of enrollment on COPE patients is captured by the interaction term C_i_*T2_k_, and its coefficient β_5_ measures the difference between COPE and non-COPE patient utilization following enrollment.

Variables X_mi_ – X_ui_ and their associated coefficients measure four socio-demographic covariates and five diagnosis covariates for each patient i that exist at the time of the intervention. The socio-demographic covariates include: age, gender, language, primary care physician. The diagnoses are: essential hypertension, major depression disorder, alcohol abuse, major cardiovascular disease (defined as at least one of the following diagnoses: acute myocardial infarction, coronary artery bypass surgery, coronary angioplasty, peripheral arterial disease, abdominal aortic aneurysm, carotid artery disease, cerebrovascular disease), and dyslipidemia.

Our primary result of interest is the value and statistical significance of the regression coefficient β_5_. The coefficient β_5_ measures the change in utilization between the control group and the COPE group following the intervention. First, if β_5_ does not differ significantly from the post-intervention slope of the control group (β_4_), then statistically there is no difference between any post-intervention change that might have occurred to the control group and the COPE group; even if some change occurred, because the control group did not experience the COPE intervention, we cannot attribute whatever change occurred in the COPE group to the COPE intervention. Second, if β_5_ is statistically significant and greater than zero, COPE patient utilization of health resources has increased relative to control group patients; if β_5_ is statistically significant and less than zero, COPE patient utilization of health resources has decreased relative to the control group.

To estimate the model described above, we used a generalized linear mixed regression model for count outcomes, assuming a Poisson distribution for the outcome variable using a log-link to assess the differences in the frequency of healthcare utilization between COPE and non-COPE patients. The analysis was implemented with the SAS PROC GLIMMIX, and performed using SAS 9.3 (SAS Institute, Cary NC).

Because the log link uses the natural log of the outcome variable to create a log-linear regression model, the regression coefficients must be exponentiated to interpret in the context of the original count data. Table 4 therefore presents the transformed regression coefficients and confidence intervals. The table accompanying this technical appendix that presents the complete regression results were not transformed so that standard errors and significance could be appropriately interpreted.

| **Technical Appendix Table 1. Percent Change in Trend of Average Healthcare Visit per Quarter by Type of Clinic Visited; 8 Quarters Pre- & Post-Intervention; Complete Model Results [1]** | | | | | | | | |
| --- | --- | --- | --- | --- | --- | --- | --- | --- |
|  |  | **Community Encounters** | | |  | **Counseling/Behavior** | | |
|  |  | **Estimate** |  |  |  | **Estimate** |  |  |
| **Variable** |  | **(SE)** | **95%CI** | **p-value** |  | **(SE)** | **95%CI** | **p-value** |
|  |  |  |  |  |  |  |  |  |
| Encounters, non-COPE patients, all covariates zero (β_0_) |  | -6.3559 | (-6.9366, -5.7752) | <.0001 |  | -2.1719 | (-2.4882, -1.8556) | <.0001 |
|  |  | 0.2963 |  |  |  | 0.1614 |  |  |
|  |  |  |  |  |  |  |  |  |
| Difference, COPE/non-COPE patients, all covariates zero (β_1_) |  | 1.8394 | (1.5591, 2.1197) | <.0001 |  | 0.5573 | (0.3576, 0.757) | <.0001 |
|  |  | 0.143 |  |  |  | 0.1019 |  |  |
|  |  |  |  |  |  |  |  |  |
| Non-COPE patient utilization trend, pre-intervention (β_2_)) |  | -0.0432 | (-0.0574, -0.0289) | <.0001 |  | 0.05 | (0.0409, 0.0591) | <.0001 |
|  |  | 0.0073 |  |  |  | 0.0046 |  |  |
|  |  |  |  |  |  |  |  |  |
| Difference, COPE versus non-COPE patient utilization trend, pre-intervention (β_3_) |  | -0.0025 | (-0.0293, 0.0243) | 0.8546 |  | -0.056 | (-0.0823, -0.0297) | <.0001 |
|  |  | 0.0137 |  |  |  | 0.0134 |  |  |
|  |  |  |  |  |  |  |  |  |
| Difference, pre-/post-intervention utilization trend, non-COPE patients (β_4_) |  | 0.0298 | (0.0029, 0.0567) | 0.0301 |  | -0.0866 | (-0.1024, -0.0708) | <.0001 |
|  |  | 0.0137 |  |  |  | 0.0081 |  |  |
|  |  |  |  |  |  |  |  |  |
| Difference, pre-/post-intervention utilization trend, COPE versus non-COPE patients (β_5_) |  | -0.004 | (-0.0553, 0.0473) | 0.8791 |  | 0.0611 | (0.0131, 0.1091) | 0.0126 |
|  |  | 0.0262 |  |  |  | 0.0245 |  |  |
|  |  |  |  |  |  |  |  |  |

| Native Language |  | 0.6362 | (0.4652, 0.8072) | <.0001 |  | 0.0989 | (0.0063, 0.1914) | 0.0363 |
| --- | --- | --- | --- | --- | --- | --- | --- | --- |
|  |  | 0.0873 |  |  |  | 0.0472 |  |  |
|  |  |  |  |  |  |  |  |  |
| Depression |  | 0.3349 | (0.0842, 0.5856) | 0.0089 |  | 0.6386 | (0.5055, 0.7717) | <.0001 |
|  |  | 0.1279 |  |  |  | 0.0679 |  |  |
|  |  |  |  |  |  |  |  |  |
| Ethyl Alcohol Abuse (ETOH) |  | 0.7302 | (0.271, 1.1894) | 0.0018 |  | 0.2446 | (-0.0129, 0.5021) | 0.0626 |
|  |  | 0.2343 |  |  |  | 0.1314 |  |  |
|  |  |  |  |  |  |  |  |  |
| Essential Hypertension |  | -0.3149 | (-0.7424, 0.1126) | 0.1489 |  | -0.1125 | (-0.3597, 0.1347) | 0.3725 |
|  |  | 0.2181 |  |  |  | 0.1261 |  |  |
|  |  |  |  |  |  |  |  |  |
| Dyslipidemia |  | -0.2123 | (-0.3855, -0.0391) | 0.0163 |  | 0.0132 | (-0.0823, 0.1087) | 0.7867 |
|  |  | 0.0884 |  |  |  | 0.0487 |  |  |
|  |  |  |  |  |  |  |  |  |
| Major Cardiovascular Disease |  | 0.5037 | (0.0556, 0.9518) | 0.0275 |  | 0.0002 | (-0.2567, 0.2572) | 0.9986 |
|  |  | 0.2286 |  |  |  | 0.1311 |  |  |
|  |  |  |  |  |  |  |  |  |
| Gender |  | -0.0816 | (-0.2579, 0.0948) | 0.3647 |  | 0.0143 | (-0.0806, 0.1093) | 0.7673 |
|  |  | 0.09 |  |  |  | 0.0485 |  |  |
|  |  |  |  |  |  |  |  |  |
| Age at time of visit |  | 0.0388 | (0.0304, 0.0472) | <.0001 |  | 0.0042 | (-0.0004, 0.0088) | 0.0758 |
|  |  | 0.0043 |  |  |  | 0.0023 |  |  |
|  |  |  |  |  |  |  |  |  |
| Primary Care Physician |  | -0.4105 | (-0.6571, -0.1639) | 0.0011 |  | 0.0487 | (-0.1034, 0.2009) | 0.5301 |
|  |  | 0.1258 |  |  |  | 0.0776 |  |  |
|  |  |  |  |  |  |  |  |  |
|  |  |  |  |  |  |  |  |  |

| **Technical Appendix Table 1. Percent Change in Trend of Average Healthcare Visit per Quarter by Type of Clinic Visited; 8 Months Pre- & Post-Intervention; Complete Model Results** | | | | | | | | |
| --- | --- | --- | --- | --- | --- | --- | --- | --- |
|  |  | **Dentist** | | |  | **Emergency** | | |
|  |  | **Estimate** |  |  |  | **Estimate** |  |  |
| **Variable** |  | **(SE)** | **95%CI** | **p-value** |  | **(Standard Error)** | **95%CI** | **p-value** |
|  |  |  |  |  |  |  |  |  |
| Encounters, non-COPE patients, all covariates equal to zero (β_0_) |  | -2.701 | (-3.0877, -2.3143) | <.0001 |  | -1.653 | (-1.9392, -1.3668) | <.0001 |
|  |  | 0.1973 |  |  |  | 0.146 |  |  |
|  |  |  |  |  |  |  |  |  |
| Difference, COPE/non-COPE patients, all covariates zero (β_1_) |  | -0.3755 | (-0.6554, -0.0956) | 0.0086 |  | 0.104 | (-0.0875, 0.2955) | 0.2872 |
|  |  | 0.1428 |  |  |  | 0.0977 |  |  |
|  |  |  |  |  |  |  |  |  |
| Non-COPE patient utilization trend, pre-intervention (β_2_)) |  | 0.0291 | (0.0207, 0.0374) | <.0001 |  | -0.0167 | (-0.0226, -0.0109) | <.0001 |
|  |  | 0.0043 |  |  |  | 0.003 |  |  |
|  |  |  |  |  |  |  |  |  |
| Difference, COPE versus non-COPE patient utilization trend, pre-intervention (β_3_) |  | -0.0166 | (-0.0572, 0.024) | 0.4236 |  | -0.017 | (-0.0384, 0.0045) | 0.1211 |
|  |  | 0.0207 |  |  |  | 0.011 |  |  |
|  |  |  |  |  |  |  |  |  |
| Difference, pre-/post-intervention utilization trend, non-COPE patients (β_4_) |  | -0.0315 | (-0.0458, -0.0172) | <.0001 |  | -0.02 | (-0.0307, -0.0093) | 0.0003 |
|  |  | 0.0073 |  |  |  | 0.0055 |  |  |
|  |  |  |  |  |  |  |  |  |
| Difference, pre-/post-intervention utilization trend, COPE versus non-COPE patients (β_5_) |  | 0.0402 | (-0.0304, 0.1108) | 0.2645 |  | 0.0072 | (-0.0337, 0.0481) | 0.729 |
|  |  | 0.036 |  |  |  | 0.0209 |  |  |
|  |  |  |  |  |  |  |  |  |
| Native Language |  | -0.1111 | (-0.2222, 0) | 0.0502 |  | 0.1028 | (0.0171, 0.1885) | 0.0187 |
|  |  | 0.0567 |  |  |  | 0.0437 |  |  |
|  |  |  |  |  |  |  |  |  |
| Depression |  | 0.1344 | (-0.027, 0.2958) | 0.1026 |  | 0.2598 | (0.1353, 0.3843) | <.0001 |
|  |  | 0.0823 |  |  |  | 0.0635 |  |  |
|  |  |  |  |  |  |  |  |  |
| Ethyl Alcohol Abuse (ETOH) |  | 0.0723 | (-0.2511, 0.3957) | 0.6612 |  | 0.6055 | (0.3728, 0.8382) | <.0001 |
|  |  | 0.165 |  |  |  | 0.1187 |  |  |
|  |  |  |  |  |  |  |  |  |
| Essential Hypertension |  | -0.2136 | (-0.5123, 0.0851) | 0.161 |  | -0.467 | (-0.6926, -0.2414) | <.0001 |
|  |  | 0.1524 |  |  |  | 0.1151 |  |  |
|  |  |  |  |  |  |  |  |  |
| Dyslipidemia |  | 0.1626 | (0.0494, 0.2758) | 0.0049 |  | -0.0124 | (-0.0989, 0.074) | 0.778 |
|  |  | 0.0578 |  |  |  | 0.0441 |  |  |
|  |  |  |  |  |  |  |  |  |
| Major Cardiovascular Disease |  | 0.3639 | (0.0534, 0.6744) | 0.0216 |  | 0.3672 | (0.1334, 0.601) | 0.0021 |
|  |  | 0.1584 |  |  |  | 0.1193 |  |  |
|  |  |  |  |  |  |  |  |  |
| Gender |  | 0.179 | (0.0629, 0.2951) | 0.0025 |  | 0.2749 | (0.1873, 0.3625) | <.0001 |
|  |  | 0.0592 |  |  |  | 0.0447 |  |  |
|  |  |  |  |  |  |  |  |  |
| Age at time of visit |  | 0.0012 | (-0.0044, 0.0067) | 0.6737 |  | 0.0034 | (-0.0008, 0.0076) | 0.1083 |
|  |  | 0.0028 |  |  |  | 0.0021 |  |  |
|  |  |  |  |  |  |  |  |  |
| Primary Care Physician |  | 0.4567 | (0.2677, 0.6457) | <.0001 |  | 0.0342 | (-0.0991, 0.1675) | 0.6146 |
|  |  | 0.0964 |  |  |  | 0.068 |  |  |
|  |  |  |  |  |  |  |  |  |
|  |  |  |  |  |  |  |  |  |

| **Technical Appendix Table 1. Percent Change in Trend of Average Healthcare Visit per Quarter by Type of Clinic Visited; 8 Months Pre- & Post-Intervention; Complete Model Results** | | | | | | | | |
| --- | --- | --- | --- | --- | --- | --- | --- | --- |
|  |  | **Inpatient** | | |  | **Primary Outpatient** | | |
|  |  | **Estimate** |  |  |  | **Estimate** |  |  |
| **Variable** |  | **(Standard Error)** | **95%CI** | **p-value** |  | **(Standard Error)** | **95%CI** | **p-value** |
|  |  |  |  |  |  |  |  |  |
| Encounters, non-COPE patients, all covariates equal to zero (β_0_) |  | -3.3979 | (-3.8383, -2.9575) | <.0001 |  | -0.9116 | (-1.1062, -0.717) | <.0001 |
|  |  | 0.2247 |  |  |  | 0.0993 |  |  |
|  |  |  |  |  |  |  |  |  |
| Difference, COPE/non-COPE patients, all covariates zero (β_1_) |  | 0.098 | (-0.1836, 0.3797) | 0.4953 |  | 0.1302 | (0.0067, 0.2537) | 0.039 |
|  |  | 0.1437 |  |  |  | 0.063 |  |  |
|  |  |  |  |  |  |  |  |  |
| Non-COPE patient utilization trend, pre-intervention (β_2_)) |  | 0.0183 | (0.0119, 0.0248) | <.0001 |  | 0.0238 | (0.0197, 0.0279) | <.0001 |
|  |  | 0.0033 |  |  |  | 0.0021 |  |  |
|  |  |  |  |  |  |  |  |  |
| Difference, COPE versus non-COPE patient utilization trend, pre-intervention (β_3_) |  | -0.0198 | (-0.0446, 0.0051) | 0.1186 |  | -0.0196 | (-0.0352, -0.004) | 0.0137 |
|  |  | 0.0127 |  |  |  | 0.008 |  |  |
|  |  |  |  |  |  |  |  |  |
| Difference, pre-/post-intervention utilization trend, non-COPE patients (β_4_) |  | -0.0145 | (-0.0255, -0.0034) | 0.0102 |  | -0.0247 | (-0.0318, -0.0177) | <.0001 |
|  |  | 0.0056 |  |  |  | 0.0036 |  |  |
|  |  |  |  |  |  |  |  |  |
| Difference, pre-/post-intervention utilization trend, COPE versus non-COPE patients (β_5_) |  | 0.0246 | (-0.0194, 0.0686) | 0.2734 |  | 0.0314 | (0.0041, 0.0587) | 0.0243 |
|  |  | 0.0225 |  |  |  | 0.0139 |  |  |
|  |  |  |  |  |  |  |  |  |
| Native Language |  | -0.1411 | (-0.2742, -0.008) | 0.0378 |  | 0.0615 | (0.0018, 0.1212) | 0.0435 |
|  |  | 0.0679 |  |  |  | 0.0305 |  |  |
|  |  |  |  |  |  |  |  |  |
| Depression |  | 0.1743 | (-0.0239, 0.3725) | 0.0847 |  | 0.1049 | (0.0182, 0.1916) | 0.0177 |
|  |  | 0.1011 |  |  |  | 0.0443 |  |  |
|  |  |  |  |  |  |  |  |  |
| Ethyl Alcohol Abuse (ETOH) |  | 0.0818 | (-0.307, 0.4707) | 0.68 |  | -0.0927 | (-0.2671, 0.0817) | 0.2974 |
|  |  | 0.1984 |  |  |  | 0.089 |  |  |
|  |  |  |  |  |  |  |  |  |
| Essential Hypertension |  | -0.4697 | (-0.8233, -0.1161) | 0.0092 |  | -0.0055 | (-0.1733, 0.1624) | 0.949 |
|  |  | 0.1804 |  |  |  | 0.0856 |  |  |
|  |  |  |  |  |  |  |  |  |
| Dyslipidemia |  | 0.2054 | (0.0708, 0.34) | 0.0028 |  | 0.0032 | (-0.0547, 0.0611) | 0.9141 |
|  |  | 0.0687 |  |  |  | 0.0296 |  |  |
|  |  |  |  |  |  |  |  |  |
| Major Cardiovascular Disease |  | 0.4978 | (0.1315, 0.8641) | 0.0077 |  | 0.151 | (-0.0209, 0.3229) | 0.0851 |
|  |  | 0.1869 |  |  |  | 0.0877 |  |  |
|  |  |  |  |  |  |  |  |  |
| Gender |  | 0.0423 | (-0.0871, 0.1717) | 0.5218 |  | 0.2275 | (0.1661, 0.2889) | <.0001 |
|  |  | 0.066 |  |  |  | 0.0313 |  |  |
|  |  |  |  |  |  |  |  |  |
| Age at time of visit |  | 0.023 | (0.0165, 0.0295) | <.0001 |  | 0.0071 | (0.0042, 0.01) | <.0001 |
|  |  | 0.0033 |  |  |  | 0.0015 |  |  |
|  |  |  |  |  |  |  |  |  |
| Primary Care Physician |  | -0.0678 | (-0.2554, 0.1199) | 0.4791 |  | 0.0777 | (-0.0042, 0.1597) | 0.0631 |
|  |  | 0.0958 |  |  |  | 0.0418 |  |  |
|  |  |  |  |  |  |  |  |  |
|  |  |  |  |  |  |  |  |  |

| **Technical Appendix Table 1. Percent Change in Trend of Average Healthcare Visit per Quarter by Type of Clinic Visited; 8 Months Pre- & Post-Intervention; Complete Model Results** | | | | | | | | |
| --- | --- | --- | --- | --- | --- | --- | --- | --- |
|  |  | **Specialty Outpatient** | | |  | **Laboratory** | | |
|  |  | **Estimate** |  |  |  | **Estimate** |  |  |
| **Variable** |  | **(Standard Error)** | **95%CI** | **p-value** |  | **(Standard Error)** | **95%CI** | **p-value** |
|  |  |  |  |  |  |  |  |  |
| Encounters, non-COPE patients, all covariates equal to zero (β_0_) |  | -2.0452 | (-2.2767, -1.8137) | <.0001 |  | -1.4648 | (-1.8249, -1.1047) | <.0001 |
|  |  | 0.1181 |  |  |  | 0.1837 |  |  |
|  |  |  |  |  |  |  |  |  |
| Difference, COPE/non-COPE patients, all covariates zero (β_1_) |  | 0.2317 | (0.0834, 0.38) | 0.0022 |  | 0.2011 | (-0.0261, 0.4283) | 0.0828 |
|  |  | 0.0756 |  |  |  | 0.1159 |  |  |
|  |  |  |  |  |  |  |  |  |
| Non-COPE patient utilization trend, pre-intervention (β_2_)) |  | 0.0177 | (0.0135, 0.022) | <.0001 |  | 0.1827 | (0.1753, 0.1901) | <.0001 |
|  |  | 0.0022 |  |  |  | 0.0038 |  |  |
|  |  |  |  |  |  |  |  |  |
| Difference, COPE versus non-COPE patient utilization trend, pre-intervention (β_3_) |  | 0.0028 | (-0.0131, 0.0186) | 0.7333 |  | -0.0196 | (-0.0468, 0.0077) | 0.1602 |
|  |  | 0.0081 |  |  |  | 0.0139 |  |  |
|  |  |  |  |  |  |  |  |  |
| Difference, pre-/post-intervention utilization trend, non-COPE patients (β_4_) |  | -0.0304 | (-0.0378, -0.0229) | <.0001 |  | -0.2221 | (-0.2332, -0.211) | <.0001 |
|  |  | 0.0038 |  |  |  | 0.0057 |  |  |
|  |  |  |  |  |  |  |  |  |
| Difference, pre-/post-intervention utilization trend, COPE versus non-COPE patients (β_5_) |  | 0.004 | (-0.024, 0.032) | 0.7807 |  | 0.0216 | (-0.0203, 0.0634) | 0.3122 |
|  |  | 0.0143 |  |  |  | 0.0214 |  |  |
|  |  |  |  |  |  |  |  |  |
| Native Language |  | -0.0453 | (-0.1125, 0.022) | 0.1874 |  | 0.1565 | (0.0444, 0.2686) | 0.0063 |
|  |  | 0.0343 |  |  |  | 0.0572 |  |  |
|  |  |  |  |  |  |  |  |  |
| Depression |  | 0.1482 | (0.0485, 0.2479) | 0.0036 |  | 0.0211 | (-0.1362, 0.1784) | 0.7925 |
|  |  | 0.0509 |  |  |  | 0.0802 |  |  |
|  |  |  |  |  |  |  |  |  |
| Ethyl Alcohol Abuse (ETOH) |  | 0.1684 | (-0.0263, 0.3631) | 0.0901 |  | 0.0324 | (-0.2732, 0.338) | 0.8354 |
|  |  | 0.0994 |  |  |  | 0.1559 |  |  |
|  |  |  |  |  |  |  |  |  |
| Essential Hypertension |  | -0.364 | (-0.5442, -0.1838) | <.0001 |  | -0.1337 | (-0.422, 0.1546) | 0.3633 |
|  |  | 0.0919 |  |  |  | 0.1471 |  |  |
|  |  |  |  |  |  |  |  |  |
| Dyslipidemia |  | 0.0327 | (-0.0362, 0.1016) | 0.3527 |  | -0.0278 | (-0.1372, 0.0816) | 0.6185 |
|  |  | 0.0352 |  |  |  | 0.0558 |  |  |
|  |  |  |  |  |  |  |  |  |
| Major Cardiovascular Disease |  | 0.4603 | (0.2731, 0.6475) | <.0001 |  | 0.1103 | (-0.1876, 0.4082) | 0.4681 |
|  |  | 0.0955 |  |  |  | 0.152 |  |  |
|  |  |  |  |  |  |  |  |  |
| Gender |  | 0.1276 | (0.0585, 0.1967) | 0.0003 |  | 0.2087 | (0.0962, 0.3212) | 0.0003 |
|  |  | 0.0353 |  |  |  | 0.0574 |  |  |
|  |  |  |  |  |  |  |  |  |
| Age at time of visit |  | 0.0201 | (0.0167, 0.0234) | <.0001 |  | 0 | (-0.0053, 0.0054) | 0.9897 |
|  |  | 0.0017 |  |  |  | 0.0027 |  |  |
|  |  |  |  |  |  |  |  |  |
| Primary Care Physician |  | 0.2828 | (0.1739, 0.3917) | <.0001 |  | 0.2053 | (0.0422, 0.3684) | 0.0136 |
|  |  | 0.0556 |  |  |  | 0.0832 |  |  |
|  |  |  |  |  |  |  |  |  |
|  |  |  |  |  |  |  |  |  |

| **Technical Appendix Table 1. Percent Change in Trend of Average Healthcare Visit per Quarter by Type of Clinic Visited; 8 Months Pre- & Post-Intervention; Complete Model Results** | | | | | | | | |
| --- | --- | --- | --- | --- | --- | --- | --- | --- |
|  |  | **Pharmacy** | | |  | **Radiology** | | |
|  |  | **Estimate** |  |  |  | **Estimate** |  |  |
| **Variable** |  | **(Standard Error)** | **95%CI** | **p-value** |  | **(Standard Error)** | **95%CI** | **p-value** |
|  |  |  |  |  |  |  |  |  |
| Encounters, non-COPE patients, all covariates equal to zero (β_0_) |  | -0.7634 | (-0.9819, -0.5449) | <.0001 |  | -4.3139 | (-4.8453, -3.7825) | <.0001 |
|  |  | 0.1115 |  |  |  | 0.2711 |  |  |
|  |  |  |  |  |  |  |  |  |
| Difference, COPE/non-COPE patients, all covariates zero (β_1_) |  | -0.0252 | (-0.1648, 0.1144) | 0.7239 |  | 0.1848 | (-0.2035, 0.5731) | 0.3509 |
|  |  | 0.0712 |  |  |  | 0.1981 |  |  |
|  |  |  |  |  |  |  |  |  |
| Non-COPE patient utilization trend, pre-intervention (β_2_)) |  | 0.1264 | (0.1206, 0.1322) | <.0001 |  | 0.0207 | (0.0009, 0.0404) | 0.0409 |
|  |  | 0.0029 |  |  |  | 0.0101 |  |  |
|  |  |  |  |  |  |  |  |  |
| Difference, COPE versus non-COPE patient utilization trend, pre-intervention (β_3_) |  | -0.0785 | (-0.0979, -0.059) | <.0001 |  | 0.0085 | (-0.0682, 0.0852) | 0.8272 |
|  |  | 0.0099 |  |  |  | 0.0391 |  |  |
|  |  |  |  |  |  |  |  |  |
| Difference, pre-/post-intervention utilization trend, non-COPE patients (β_4_) |  | -0.2141 | (-0.2237, -0.2045) | <.0001 |  | -0.0129 | (-0.0463, 0.0205) | 0.45 |
|  |  | 0.0049 |  |  |  | 0.017 |  |  |
|  |  |  |  |  |  |  |  |  |
| Difference, pre-/post-intervention utilization trend, COPE versus non-COPE patients (β_5_) |  | 0.0863 | (0.0519, 0.1206) | <.0001 |  | 0.0135 | (-0.1111, 0.138) | 0.8321 |
|  |  | 0.0176 |  |  |  | 0.0635 |  |  |
|  |  |  |  |  |  |  |  |  |
| Native Language |  | -0.0451 | (-0.1085, 0.0182) | 0.1626 |  | 0.0159 | (-0.1381, 0.1699) | 0.8393 |
|  |  | 0.0323 |  |  |  | 0.0786 |  |  |
|  |  |  |  |  |  |  |  |  |
| Depression |  | 0.0595 | (-0.0459, 0.165) | 0.2686 |  | 0.2161 | (-0.0095, 0.4417) | 0.0605 |
|  |  | 0.0538 |  |  |  | 0.1151 |  |  |
|  |  |  |  |  |  |  |  |  |
| Ethyl Alcohol Abuse (ETOH) |  | 0.0596 | (-0.1079, 0.2271) | 0.4856 |  | -0.2596 | (-0.7735, 0.2543) | 0.3221 |
|  |  | 0.0855 |  |  |  | 0.2622 |  |  |
|  |  |  |  |  |  |  |  |  |
| Essential Hypertension |  | -0.0368 | (-0.1956, 0.122) | 0.6495 |  | 0.0038 | (-0.4076, 0.4152) | 0.9856 |
|  |  | 0.081 |  |  |  | 0.2099 |  |  |
|  |  |  |  |  |  |  |  |  |
| Dyslipidemia |  | -0.1306 | (-0.197, -0.0642) | 0.0001 |  | -0.2147 | (-0.3704, -0.059) | 0.0069 |
|  |  | 0.0339 |  |  |  | 0.0795 |  |  |
|  |  |  |  |  |  |  |  |  |
| Major Cardiovascular Disease |  | 0.037 | (-0.1298, 0.2038) | 0.6639 |  | -0.0893 | (-0.5152, 0.3366) | 0.6811 |
|  |  | 0.0851 |  |  |  | 0.2173 |  |  |
|  |  |  |  |  |  |  |  |  |
| Gender |  | 0.0402 | (-0.0226, 0.103) | 0.2102 |  | 0.5288 | (0.3612, 0.6964) | <.0001 |
|  |  | 0.0321 |  |  |  | 0.0855 |  |  |
|  |  |  |  |  |  |  |  |  |
| Age at time of visit |  | 0.0071 | (0.0039, 0.0103) | <.0001 |  | 0.0085 | (0.0008, 0.0161) | 0.0304 |
|  |  | 0.0016 |  |  |  | 0.0039 |  |  |
|  |  |  |  |  |  |  |  |  |
| Primary Care Physician |  | 0.1491 | (0.0418, 0.2564) | 0.0064 |  | -0.2393 | (-0.4784, -0.0002) | 0.0499 |
|  |  | 0.0547 |  |  |  | 0.122 |  |  |
|  |  |  |  |  |  |  |  |  |
|  |  |  |  |  |  |  |  |  |

| **Technical Appendix Table 1. Percent Change in Trend of Average Healthcare Visit per Quarter by Type of Clinic Visited; 8 Months Pre- & Post-Intervention; Complete Model Results** | | | | | | | | |
| --- | --- | --- | --- | --- | --- | --- | --- | --- |
|  |  | **Total 1 (all clinic utilization, excluding laboratory, pharmacy & radiology)** | | |  | **Total 2 (Total 1, excluding**  **Community Encounters)** | | |
|  |  | **Estimate** |  |  |  | **Estimate** |  |  |
| **Variable** |  | **(Standard Error)** | **95%CI** | **p-value** |  | **(Standard Error)** | **95%CI** | **p-value** |
|  |  |  |  |  |  |  |  |  |
| Encounters, non-COPE patients, all covariates equal to zero (β_0_) |  | -0.0606 | (-0.2092, 0.088) | 0.4239 |  | -0.0147 | (-0.163, 0.1336) | 0.846 |
|  |  | 0.0758 |  |  |  | 0.0757 |  |  |
|  |  |  |  |  |  |  |  |  |
| Difference, COPE/non-COPE patients, all covariates zero (β_1_) |  | 0.2111 | (0.1171, 0.3051) | <.0001 |  | 0.1303 | (0.0361, 0.2245) | 0.0068 |
|  |  | 0.048 |  |  |  | 0.0481 |  |  |
|  |  |  |  |  |  |  |  |  |
| Non-COPE patient utilization trend, pre-intervention (β_2_)) |  | 0.0104 | (0.0081, 0.0126) | <.0001 |  | 0.012 | (0.0097, 0.0143) | <.0001 |
|  |  | 0.0012 |  |  |  | 0.0012 |  |  |
|  |  |  |  |  |  |  |  |  |
| Difference, COPE versus non-COPE patient utilization trend, pre-intervention (β_3_) |  | -0.0225 | (-0.0305, -0.0145) | <.0001 |  | -0.0168 | (-0.0252, -0.0083) | 0.0001 |
|  |  | 0.0041 |  |  |  | 0.0043 |  |  |
|  |  |  |  |  |  |  |  |  |
| Difference, pre-/post-intervention utilization trend, non-COPE patients (β_4_) |  | -0.0172 | (-0.0211, -0.0133) | <.0001 |  | -0.019 | (-0.023, -0.015) | <.0001 |
|  |  | 0.002 |  |  |  | 0.002 |  |  |
|  |  |  |  |  |  |  |  |  |
| Difference, pre-/post-intervention utilization trend, COPE versus non-COPE patients (β_5_) |  | 0.0246 | (0.0102, 0.039) | 0.0008 |  | 0.0203 | (0.0052, 0.0354) | 0.0086 |
|  |  | 0.0073 |  |  |  | 0.0077 |  |  |
|  |  |  |  |  |  |  |  |  |
| Native Language |  | 0.0124 | (-0.0318, 0.0566) | 0.5827 |  | -0.0026 | (-0.0466, 0.0414) | 0.9077 |
|  |  | 0.0225 |  |  |  | 0.0225 |  |  |
|  |  |  |  |  |  |  |  |  |
| Depression |  | 0.1518 | (0.0881, 0.2155) | <.0001 |  | 0.1543 | (0.0907, 0.2179) | <.0001 |
|  |  | 0.0325 |  |  |  | 0.0324 |  |  |
|  |  |  |  |  |  |  |  |  |
| Ethyl Alcohol Abuse (ETOH) |  | 0.1883 | (0.0642, 0.3124) | 0.003 |  | 0.1708 | (0.0469, 0.2947) | 0.0069 |
|  |  | 0.0633 |  |  |  | 0.0632 |  |  |
|  |  |  |  |  |  |  |  |  |
| Essential Hypertension |  | -0.2496 | (-0.3681, -0.1311) | <.0001 |  | -0.2557 | (-0.3739, -0.1375) | <.0001 |
|  |  | 0.0605 |  |  |  | 0.0603 |  |  |
|  |  |  |  |  |  |  |  |  |
| Dyslipidemia |  | 0.0433 | (-0.0017, 0.0884) | 0.0594 |  | 0.0472 | (0.0023, 0.0921) | 0.0395 |
|  |  | 0.023 |  |  |  | 0.0229 |  |  |
|  |  |  |  |  |  |  |  |  |
| Major Cardiovascular Disease |  | 0.317 | (0.1945, 0.4395) | <.0001 |  | 0.3246 | (0.2025, 0.4467) | <.0001 |
|  |  | 0.0625 |  |  |  | 0.0623 |  |  |
|  |  |  |  |  |  |  |  |  |
| Gender |  | 0.1544 | (0.11, 0.1988) | <.0001 |  | 0.1559 | (0.1116, 0.2002) | <.0001 |
|  |  | 0.0226 |  |  |  | 0.0226 |  |  |
|  |  |  |  |  |  |  |  |  |
| Age at time of visit |  | 0.0142 | (0.012, 0.0163) | <.0001 |  | 0.0131 | (0.0109, 0.0153) | <.0001 |
|  |  | 0.0011 |  |  |  | 0.0011 |  |  |
|  |  |  |  |  |  |  |  |  |
| Primary Care Physician |  | 0.0893 | (0.0208, 0.1577) | 0.0106 |  | 0.0973 | (0.0288, 0.1658) | 0.0053 |
|  |  | 0.0349 |  |  |  | 0.0349 |  |  |
|  |  |  |  |  |  |  |  |  |
| [1] Untransformed regression coefficients from GLMR on count data using a log-link. Outcome variables are assumed to follow a Poisson distribution. | | | | | | | | |
